# Arterial spin labeling-ASPECTS and a conventional MRI-based nomogram for predicting prognosis after surgical revascularization in Moyamoya disease

**DOI:** 10.3389/fneur.2025.1649224

**Published:** 2025-12-16

**Authors:** Tao Yuan, Zhenhua Xu, Yawu Liu, Lijuan Gao, Lei Lou, Lina Geng, Haiying Cui, Guanmin Quan

**Affiliations:** 1 Department of Medical Imaging, The Second Hospital of Hebei Medical University, Shijiazhuang, China; 2 Department of Clinical Radiology, Kuopio University Hospital, Kuopio, Finland; 3 Department of Neurology, Universiity of Eastern Finland, Kuopio, Finland; 4 Department of Medical Imaging, The First Hospital of Hebei Medical University, Shijiazhuang, China

**Keywords:** Moyamoya disease, MRI, arterial spin-labeling, nomogram, prediction

## Abstract

**Objective:**

This study aimed to explore the significant factors of prognosis in patients with Moyamoya disease (MMD) after surgical revascularization and to develop a nomogram model for predicting poor prognosis.

**Materials and methods:**

We retrospectively analyzed magnetic resonance imaging (MRI) and clinical data of 128 patients with MMD. The patients were randomly assigned to training and validation cohorts in a ratio of 7:3. Multivariate logistic regression analysis was applied to identify factors significantly associated with prognosis. The predictive efficiencies of the models were evaluated using receiver operating characteristic (ROC) curves and compared using the Delong test. We then developed a nomogram model for prediction and verified it using a validation cohort.

**Results:**

Preoperative arterial spin labeling (ASL)-Alberta Stroke Program Early computed tomography Score (ASL-ASPECTS), admission modified Rankin scale (mRS) score, ivy sign, and Houkin’s grade >2 were significantly associated with poor prognosis (mRS > 2). The areas under the curves (AUCs) for predicting poor prognosis were 0.772, 0.855, 0.899, and 0.994 for clinical, conventional MRI, ASL-based, and combination models, respectively. The results of the Delong test demonstrated the superior prediction ability of the combination model compared with the clinical, conventional MRI, and ASL models (all *p* < 0.001). Calibration curve analysis showed that the predictive probability of the nomogram model was highly consistent in the training cohort. The decision curve showed a net predictive benefit in the validation cohort.

**Conclusion:**

Preoperative ASL-ASPECTS, admission mRS, ivy sign, and Houkin grade >2 were significantly associated with poor prognosis in patients with MMD after surgical revascularization. The nomogram model, including enrolled ASL-ASPECTS and MRI features, may help improve prognosis prediction.

## Introduction

Moyamoya disease (MMD) is a rare cerebrovascular disease characterized by chronic, progressive stenosis or occlusion of the distal internal carotid arteries, accompanied by the development of an abnormal vascular network at the base of the brain (1). MMD prevalence is higher in East Asia, with an annual incidence of 1.01/100,000 in China (2, 3). Since recurrent cerebral ischemic or hemorrhagic events caused by MMD seriously affect prognosis (4), the identification of factors associated with prognosis and accurate outcome prediction are critical for developing individualized therapy strategy, optimizing perioperative management, and improving long-term outcomes.

At present, the prognosis prediction of MMD mainly depends on conventional vascular imaging and clinical features (5). The classification and staging methods, such as Suzuki staging, are mainly based on angiographic findings. While these methods reflect the severity of cerebral artery stenoses, they cannot quantitatively assess downstream cerebral parenchymal blood perfusion. Meanwhile, as an invasive technique and radiation exposure, digital subtraction angiography is only employed before and during interventional procedures (4–6). Additionally, MMD prognosis is related to superimposed acute ischemic insults, including hemorrhage and brain parenchymal atrophy. Conventional computed tomography (CT) and magnetic resonance imaging (MRI) scans can provide anatomic information but are insufficient for evaluating blood perfusion. From a clinical perspective, the symptoms of MMD are complex and diverse. As a result, the pathological and pathophysiological abnormalities that influence prognosis cannot be fully understood based solely on clinical symptoms and signs. Furthermore, there remains a lack of comprehensive evaluation frameworks that integrate clinical factors and imaging data to objectively and accurately predict poor functional outcomes following surgical revascularization in patients with MMD.

Arterial spin labeling (ASL) is a noninvasive technique for evaluating cerebral blood flow (CBF) that uses magnetically labeled blood in the neck region as an endogenous tracer. It has been widely applied in evaluating cerebrovascular diseases, including Moyamoya disease (MMD) (7). Meanwhile, the Alberta Stroke Program Early CT score (ASPECTS) offers a convenient semi-quantitative method for assessing early ischemic changes in the middle cerebral artery area in patients with MMD (8). However, studies exploring the correlation between ASL-based ASPECTS and clinical outcomes in MMD remain limited.

We hypothesized that the ASPECTS derived from ASL (ASL-ASPECTS), combined with vascular signs on conventional MRI, could offer a more comprehensive assessment of CBF status in patients with MMD following revascularization, thereby improving the prediction of neurological outcomes. To test this hypothesis, we collected clinical variables, conventional MRI factors, and ASL data from patients with MMD. We then constructed a nomogram to estimate the probability of neurological deficits at 1 year after bypass surgery. This study aimed to establish an objective ASL-based method for accurately predicting postoperative prognosis in patients with MMD.

## Methods

### Study enrollment and clinical data

This study retrospectively enrolled consecutive patients diagnosed with MMD with complete clinical and imaging data between October 2022 and January 2024. The inclusion criteria were patients: (1) with a confirmed diagnosis of MMD according to the Chinese expert consensus on the treatment of MMD (9) and (2) who underwent superficial temporal artery bypass treatment. The exclusion criteria were patients: (1) with one of the following concurrent diseases: autoimmune disease, brain tumor, or cerebrovascular malformation (9); (2) with poor imaging owing to motion artifacts or incomplete sequences (10); and (3) lost to follow-up during the study period.

Clinical data on sex, age, and cerebrovascular disease-related risk factors (hypertension, diabetes, coronary heart disease, hyperlipidemia, smoking, and alcohol consumption) were obtained from patient medical records (11). Perioperative antiplatelet therapy (aspirin 100 mg/day) was routinely given to all patients; anticoagulants were avoided due to bleeding risk. Postoperative prognosis was evaluated at 12 months after bypass procedure using the modified Rankin scale score (mRS), with scores ranging from 0 to 6. The prognosis for bilateral bypass cases was evaluated after the second surgery, used as the baseline time point. According to the mRS, all patients with MMD were divided into good (mRS score 0–2) and poor (mRS score 3–6) prognosis groups (12). All patients were randomly divided into training and validation cohorts in a ratio of 7:3. The clinical and imaging factors between the two cohorts, and between good and poor prognoses were compared.

### MRI

MRI examinations were performed on a 3-T MR scanner (GE Architect 3.0, GE Healthcare, Waukesha, WI, United States) with a 48-channel head coil. The routine MRI protocol included T2-based fluid attenuation inversion recovery (T2-FLAIR; TR: 7500 ms, TE: 120 ms, TI 2227 ms), three-dimensional time-of-flight (TOF) magnetic resonance angiography (MRA, repetition time [TR]: 22 ms; echo time [TE]: 3.4 ms). Two-post-labeling delay (PLD) ASL was simulated with the following parameters: TR: 5,816 ms, TE: 10.7 ms, PLD 1–2 = 1.5 s and 2.5 s, thickness: 4.0 mm, layers: 72, and scan duration: 3 min 48 s. All MRI, including ASL perfusion, was performed at two fixed points: 1 week before and 1 week after bypass procedure.

### Image analysis

Imaging data were transferred to a workstation (GE AW4.7) and post-processing was performed using the Funtool software. Quantitative and semi-quantitative analysis imaging items included CBF and its derived metrics, white matter hyperintensity (WMH), ivy sign, and MRA grading. Both ischemic and hemorrhagic onsets were determined from MRI at first presentation. Hemorrhagic lesions had resolved before ASL-ASPECTS measurements, ensuring that scoring was not confounded.

CBF values were measured using region-of-interest (ROI) analysis in the gray matter regions within the anterior and middle cerebral artery territories of both hemispheres. Specifically, ROIs were placed in the frontal lobe, temporal lobe, insular lobe, and basal ganglia. Each ROI was measured three times, and the average value was recorded as the final CBF measurement. The relative CBF (rCBF) and change in relative CBF (ΔrCBF) were calculated using the Formulas 1, 2 (13):


rCBF=(CBFoperated side/CBFcontralateral side) (1)

ΔrCBF=(rCBF1wpostoperatively−rCBFpreoperatively) (2)

The ASPECTS score for patients with MMD was calculated using ASL-derived CBF maps. Briefly, scoring began at 10 points, with one point subtracted for each MCA region showing signs of ischemia, defined as CBF < 30 mL/100 g/min. Thus, the maximum score of 10 indicated no ischemic regions, while the minimum score of 0 indicated ischemia in all 10 MCA regions. ASPECTS was assessed at two anatomical levels on the CBF maps: (1) the basal ganglia level, including the caudate nucleus, internal capsule, insula, and M1–M3 regions; and (2) the corona radiata level, including the M4–M6 regions (4, 14). The ASL-ASPECTS score was derived by subtracting one point for each region exhibiting hypoperfusion (CBF < 30 mL/100 g/min) to quantify the extent of perfusion deficits in the MCA territory (15).

Brain white matter hyperintensities (WMH) on T2-FLAIR images were evaluated using the Fazekas method for grading. In brief, the Fazekas scale is a four-level method. The range of white matter hyperintensity lesions in the paraventricular and subcortical areas of the brain ranges from 0 to 3 (0, no white matter lesion; 1, single lesions; 2, numerous lesions; 3, confluent lesions) (16).

Ivy sign was defined as punctate or continuous linear hyperintensities in the subarachnoid space and cortical sulci on T2-FLAIR images. The ivy sign of each hemisphere was classified as grade 0–2 (0, no ivy sign; 1, less than half of the cortical surface; 2, at least half of the cortical surface) (8). The nomogram developed in the present study classified ivy sign as mild (grades 0–1) or severe (grade 2).

MRA grading in patients with MMD was performed using Houkin’s method, with each cerebral hemisphere evaluated separately. The internal carotid artery (ICA) and middle cerebral artery (MCA) were each graded from 0 to 3 (0, normal; 3, invisible), while the anterior cerebral artery (ACA) and posterior cerebral artery (PCA) were graded from 0 to 2 (0, normal; 2, invisible). The total MRA score for each patient was calculated by summing the scores from the four main cerebral arteries, yielding a possible range of 0 to 10. Based on the total score, MRA grade was classified into four categories: grade 1 (score 0–1), grade 2 (score 2–4), grade 3 (score 5–7), and grade 4 (score 8–10). The cerebral artery state was defined as “fine” for grades 1–2 and “poor” for grades 3–4 (17).

All MRI findings were independently analyzed by two neuroradiologists with 5 and 10 years of experience in neuroradiology, respectively. Any disagreements were resolved by consulting another neuroradiologist with 32 years of experience in neuroimaging to reach a consensus.

### Statistical analyses

Statistical analysis was conducted using IBM SPSS Statistics for Windows, version 27.0, MSTATA,^1^ and R (version 4.2.2, R Foundation for Statistical Computing, Vienna). The threshold for statistical significance was set at *p* < 0.05. Normally distributed data were expressed as means ± standard deviation. Non-normal data are presented as medians (interquartile range, IQR). In the univariate analysis, categorical variables were compared using chi-square or Fisher’s exact tests, whereas continuous variables were analyzed using Student’s *t*-test or Wilcoxon Mann–Whitney test. Univariate and multivariate logistic regression analyses were used to identify significant factors associated with poor postoperative outcomes in patients with MMD. The collinearity of significant factors was analyzed by SPSS Software, and the metrics was variance inflation factor (VIF).

The nomogram for predicting poor prognosis in patients with MMD was constructed using R software. Four models were developed: a clinical model, a conventional MRI model, an ASL-based model, and a combined mode, each incorporating the significant factors (*p* < 0.05). Receiver operating characteristic (ROC) was used to assess the diagnostic performance of the outcome prediction models. The model performances were compared using the Delong test. The predictive accuracy of the nomogram was further evaluated using ROC and calibration curve analysis. Additionally, decision curve analysis (DCA) was performed to evaluate the net benefit of the prediction models. Ten-fold cross-validation for all MMD patients to strengthen the model’s generalizability.

## Results

### Patient population

**Figure 1:**
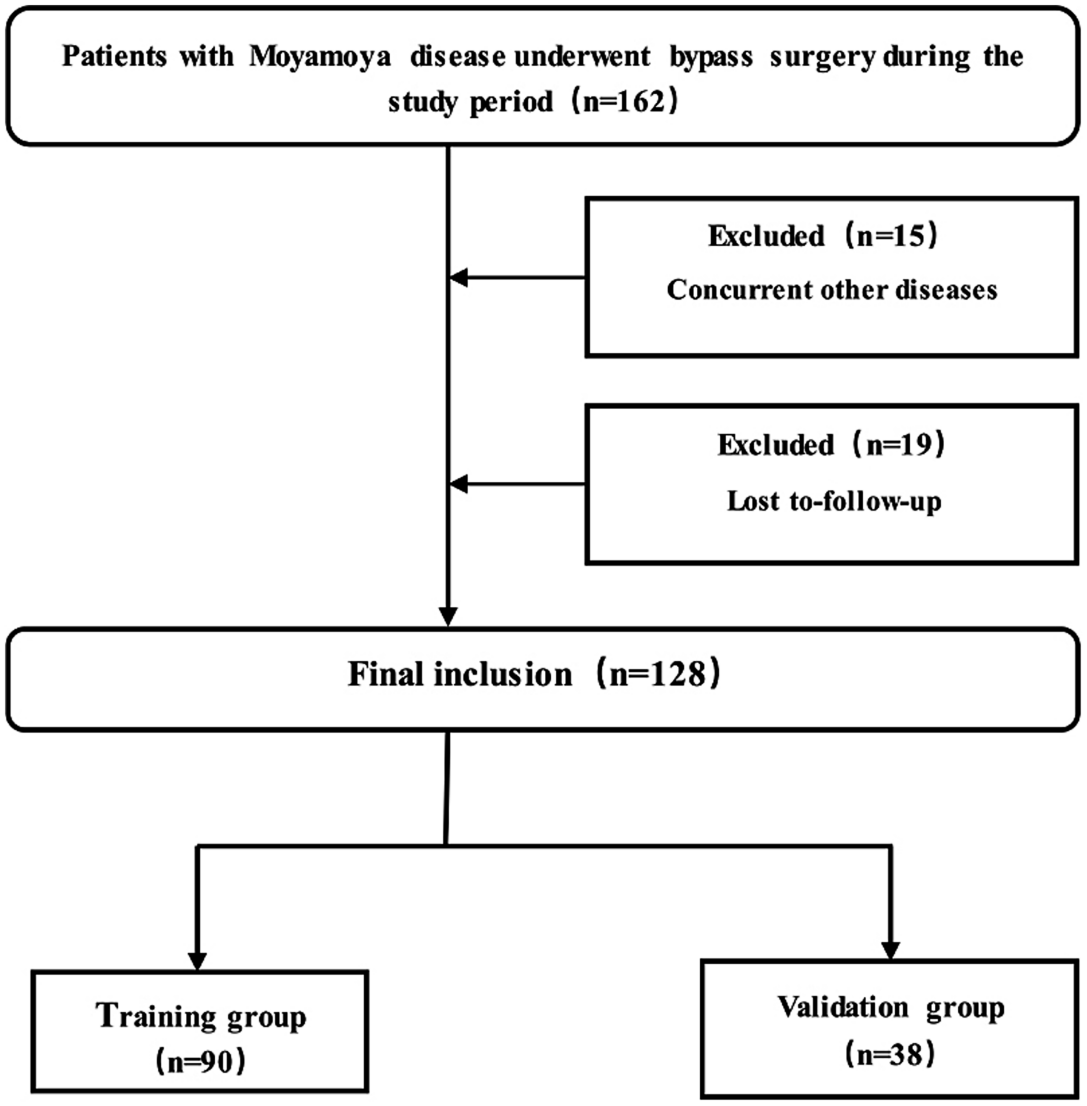
Flowchart illustrating the inclusion and exclusion of eligible patients in this study.

Among the initial 162 patients with MMD who underwent surgical revascularization, 34 were excluded for comorbidities (*n* = 15) and loss to follow-up (*n* = 19). A total of 128 patients (72 male, 56 female; mean age 46 ± 12 years) were ultimately included, comprising 3 pediatric cases (aged 5, 5, and 8 years) who all presented with ischemic onset and had good prognoses at one-year follow-up. The patients were randomly assigned to the training (*n* = 90) and validation (*n* = 38) cohorts (Figure 1). During follow-up, 56 patients (44%) had a poor prognosis, including 35 and 21 patients in training and validation cohorts, respectively. The basic clinical characteristics and imaging factors did not differ significantly between the training and validation cohorts (*p* > 0.05) (Table 1).

**Table 1:** Comparison of basic information of MMD patients with bypass treatment.

Variables	Training cohort (*n* = 90)	Validation cohort (*n* = 38)	*p* value
Gender (male)	50 (55.6%)	22 (57.9%)	0.807
Age (year-old)	46 ± 11	44 ± 12	0.397
Hypertension	53 (58.9%)	22 (57.9%)	0.917
Diabetes mellitus	19 (21.1%)	11 (28.9%)	0.339
Coronary heart disease	13 (14.4%)	8 (21.1%)	0.356
Hyperlipidemia	10 (11.1%)	8 (21.1%)	0.139
Smoke	23 (25.6%)	8 (21.1%)	0.587
Drinking alcohol	26 (28.9%)	10 (26.3%)	0.767
Onset			0.075
Bleeding	12 (13.3%)	10 (26.3%)	
Ischemia	78 (86.7%)	28 (73.7%)	
Surgery			0.920
Unilatera	56 (62.2%)	24 (63.2%)	
Bilateral	34 (37.8%)	14 (36.8%)	
Preoperative ASL-ASPECTS scores	6.00 (4.00, 7.00)	6.00 (4.00, 7.00)	0.392
Admission mRS score	2.00 (2.00, 3.00)	2.00 (2.00, 2.75)	0.485
Preoperative bleeding/infarction	31 (34.4%)	20 (52.6%)	0.055
White matter hyperintensity	2.00 (1.00, 2.00)	2.00 (1.00, 2.75)	0.133
Ivy sign	55 (61.1%)	25 (65.8%)	0.617
Houkin grading	40 (44.4%)	21 (55.3%)	0.263
rCBF 1 week before surgery	0.94 ± 0.81	0.79 ± 0.56	0.240
rCBF 1 week after surgery	1.54 ± 1.79	1.16 ± 0.62	0.080
△rCBF	0.60 ± 1.75	0.37 ± 0.63	0.277
Prognosis (poor) at 12 months after bypass	35 (38.9%)	21 (55.3%)	0.088

MMD, Moyamoya disease; ASL-ASPECTS, arterial spin labeling (ASL)-Alberta stroke program early CT score; CBF, cerebral blood flow; rCBF = (CBF_operated side_/CBF_contralateral_); △rCBF = (rCBF_1w after operation_-rCBF_before operation_).

### Comparison between the good and poor prognosis groups in the training cohort

Compared with patients with good prognosis, patients with poor outcomes showed a higher rate of hypertension (80% vs. 45%, *p* = 0.002), lower pre-operative ASL-ASPECTS score (4 vs. 7, *p* < 0.001), higher admission mRS score (3 vs. 2, *p* < 0.001), higher hemorrhage or infarction rate (57% vs. 20%, *p* < 0.001), higher WMH score (2 vs. 1, *p* < 0.001), higher rate of ivy sign (89% vs. 44%, *p* < 0.001), and higher rate of Houkin’s grade >2 (74% vs. 25%, *p* < 0.001). Sex, age, diabetes, coronary heart disease, and hyperlipidemia did not differ significantly between the groups (*p* = 0.368–0.999) (Table 2).

**Table 2:** Comparison of clinical imaging factors of MMD patients in training group with different prognoses.

Features	Good prognosis (*n* = 55)	Poor prognosis (*n* = 35)	*P* value
Gender (male)	29 (53%)	21 (60%)	0.498
Age (year-old)	47 ± 13	46 ± 9	0.631
Hypertension	25 (45%)	28 (80%)	0.001
Diabetes mellitus	10 (18%)	9 (26%)	0.393
Coronary heart disease	8 (15%)	5 (14%)	0.973
Hyperlipidemia	6 (11%)	4 (11%)	>0.999
Smoke	15 (27%)	8 (23%)	0.640
Drinking alcohol	15 (27%)	11 (31%)	0.672
Onset			0.759
Bleeding	8 (15%)	4 (11%)	
Ischemia	47 (85%)	31 (89%)	
Surgery			0.586
Unilatera	33 (60%)	23 (66%)	
Bilateral	22 (40%)	12 (34%)	
Preoperative ASL-ASPECTS scores	7.00 (6.00, 8.00)	4.00 (4.00, 5.00)	<0.001
Admission mRS score	2.00 (1.50, 2.00)	3.00 (2.00, 3.00)	<0.001
Preoperative bleeding/infarction	11 (20%)	20 (57%)	<0.001
White matter hyperintensity	1.00 (1.00, 2.00)	2.00 (1.00, 2.50)	<0.001
Ivy sign	24 (44%)	31 (89%)	<0.001
Houkin grading	14 (25%)	26 (74%)	<0.001
rCBF 1 week before surgery	0.93 ± 0.66	0.95 ± 1.02	0.931
rCBF 1 week after surgery	1.38 ± 1.39	1.80 ± 2.29	0.328
△rCBF	0.44 ± 1.60	0.85 ± 1.97	0.308

MMD, Moyamoya disease; ASL-ASPECTS, arterial spin labeling (ASL)-Alberta stroke program early CT score; CBF, cerebral blood flow; rCBF = (CBF_operated side_/CBF_contralateral_); △rCBF = (rCBF_1w after operation_-rCBF_before operation_).

There was no significant difference of the changes of mRS, (ΔmRS = mRS_pretreatment_-mRS_post-treatment_) between training and validation cohorts (1.00 vs. 0.50, *p* = 0.813) (Table 1), neither between good prognosis and poor prognosis groups (1.00 vs. 0.00, *p* = 0.339) (Table 2).

### Clinical and imaging features related to poor prognosis

The univariate logistic regression analysis revealed hypertension (odds ratio [OR] = 4.80, *p* = 0.002), preoperative ASL-ASPECTS score (OR = 0.27, *p* < 0.001), admission mRS score (OR = 4.50, *p* < 0.001), preoperative hemorrhage/infarction (OR = 5.33, *p* < 0.001), WMH (OR = 3.83, *p* < 0.001), ivy sign (OR = 0.10, *p* < 0.001), Houkin’s grade <2 (OR = 0.12, *p* < 0.001) as risk factors for poor prognosis in patients with MMD. In multivariate logistic regression analysis, preoperative ASL-ASPECTS score (OR = 0.50, *p* = 0.032), admission mRS score (OR = 2.91, *p* < 0.042), ivy sign (OR = 0.17, *p* < 0.049), and Houkin’s grade (OR = 0.14, *p* < 0.041) remained independent risk factors for poor prognosis (Table 3). The VIF values of ivy sign was 1.160, and Houkin’s grade 1.180, preoperative ASL-ASPECTS score 1.331, admission mRS score 1.153.

**Table 3:** Univariate and multivariate logistic regression analysis of significant factors of poor prognosis in MMD patients.

Variable analysis	Characteristics	OR	95% CI	*P* value
Univariate analysis	Hypertension	4.80	1.79, 12.84	0.002
	Preoperative ASL-ASPECTS scores	0.27	0.16, 0.45	<0.001
	Admission mRS score	4.50	2.18, 9.27	<0.001
	Preoperative bleeding/infarction	5.33	2.08, 13.66	<0.001
	White matter hyperintensity	3.83	1.88, 7.79	<0.001
	Ivy sign	0.10	0.03, 0.32	<0.001
	Houkin grading	0.12	0.04, 0.31	<0.001
Multivariate analysis	Preoperative ASL-ASPECTS scores	0.50	0.27, 0.94	0.032
	Admission mRS score	2.91	1.04, 8.16	0.042
	Ivy sign	0.17	0.03, 0.99	0.049
	Houkin grading	0.14	0.02, 0.92	0.041

MMD, Moyamoya disease; OR, Odds ratio; ASL-ASPECTS, arterial spin labeling (ASL)-Alberta stroke program early CT score.

### Prediction of poor prognosis

**Figure 2:**
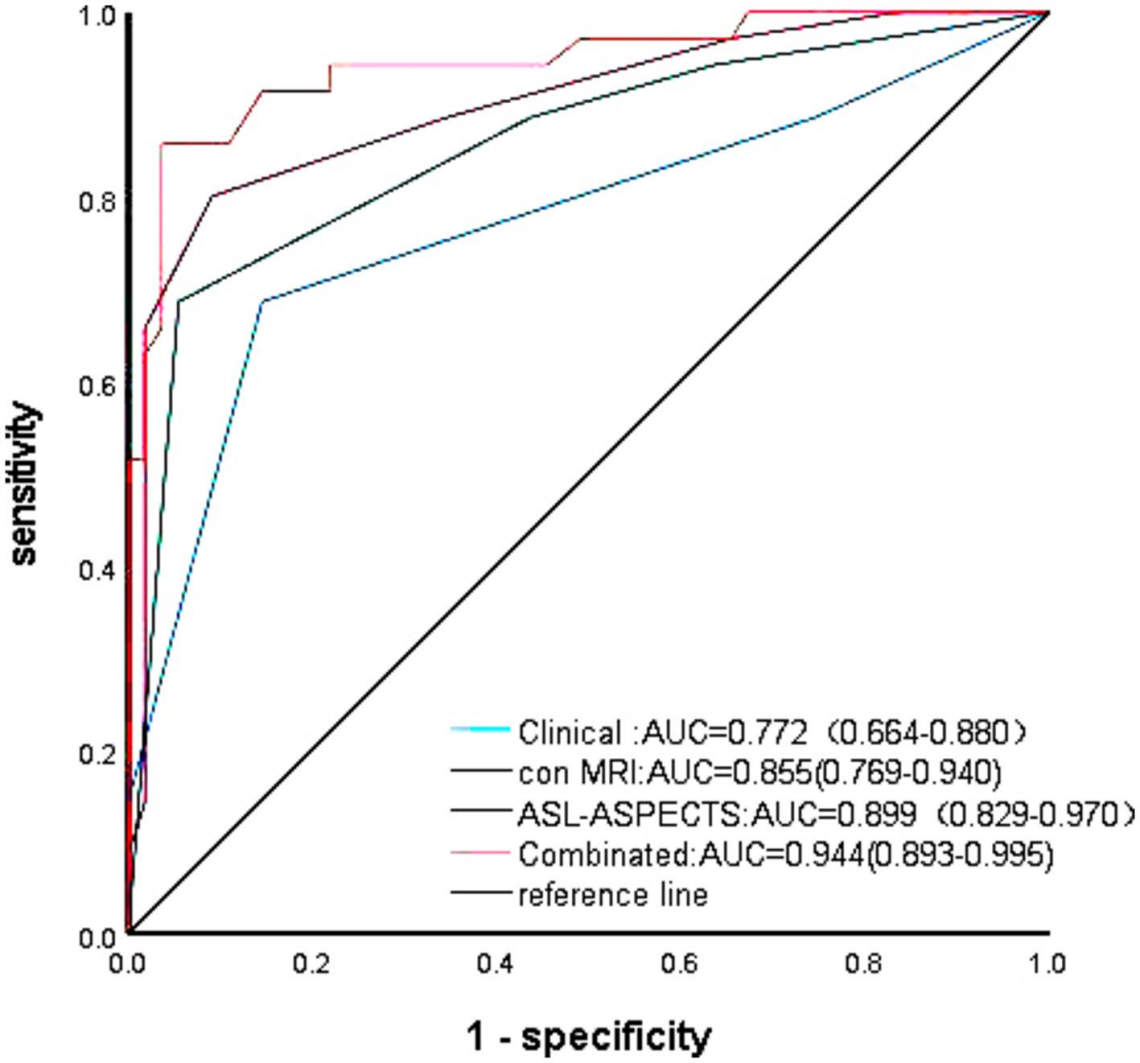
ROC analysis of various models for predicting the risk of poor prognosis in MMD patients after bypass treatment.

The prediction models included significant factors identified in the multivariate logistic regression analysis. The area under the ROC curve (AUC) for predicting poor prognosis of the clinical model, which included preoperative mRS score, was 0.772. The AUC of the conventional MRI model (Houkin’s grading+ ivy sign) was 0.855, while that for the ASL model (pre-operative ASL-ASPECTS) was 0.899. The AUC for the combined model (all significant factors) was 0.944 (Figure 2). The Delong test showed the higher predictive efficacy of the combined model compared with the clinical (*p* < 0.001), conventional MRI (*p* < 0.001), and ASL (*p* = 0.001) models. However, the predictive efficacy did not differ between the clinical and conventional MRI models (*p* = 0.587), clinical and ASL models (*p* = 0.207), and conventional MRI and ASL models (*p* = 0.459).

### Development of the nomogram prediction model

**Figure 3:**
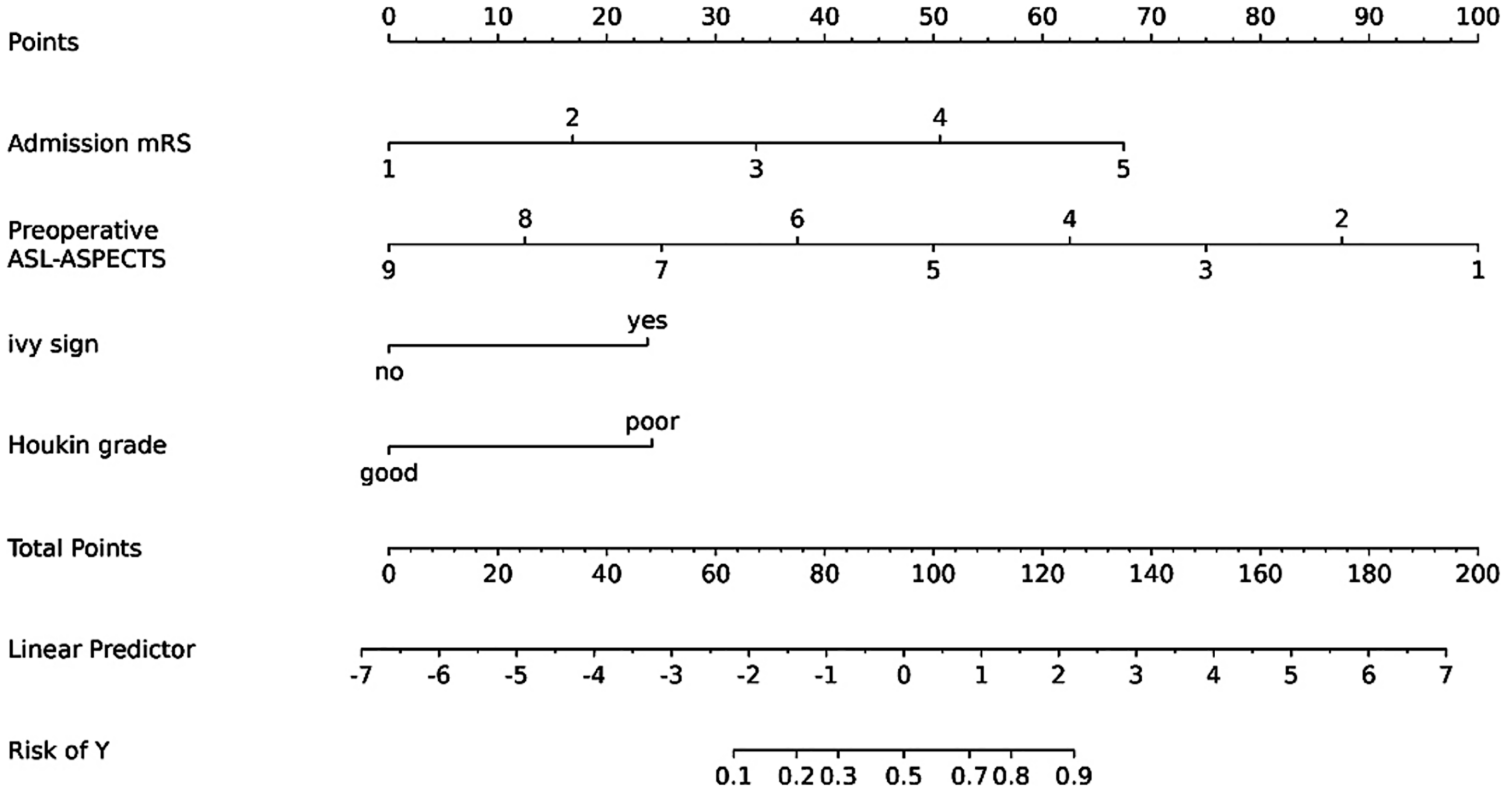
Nomogram for predicting the poor prognosis of MMD patients after bypass treatment.

**Figure 4:**
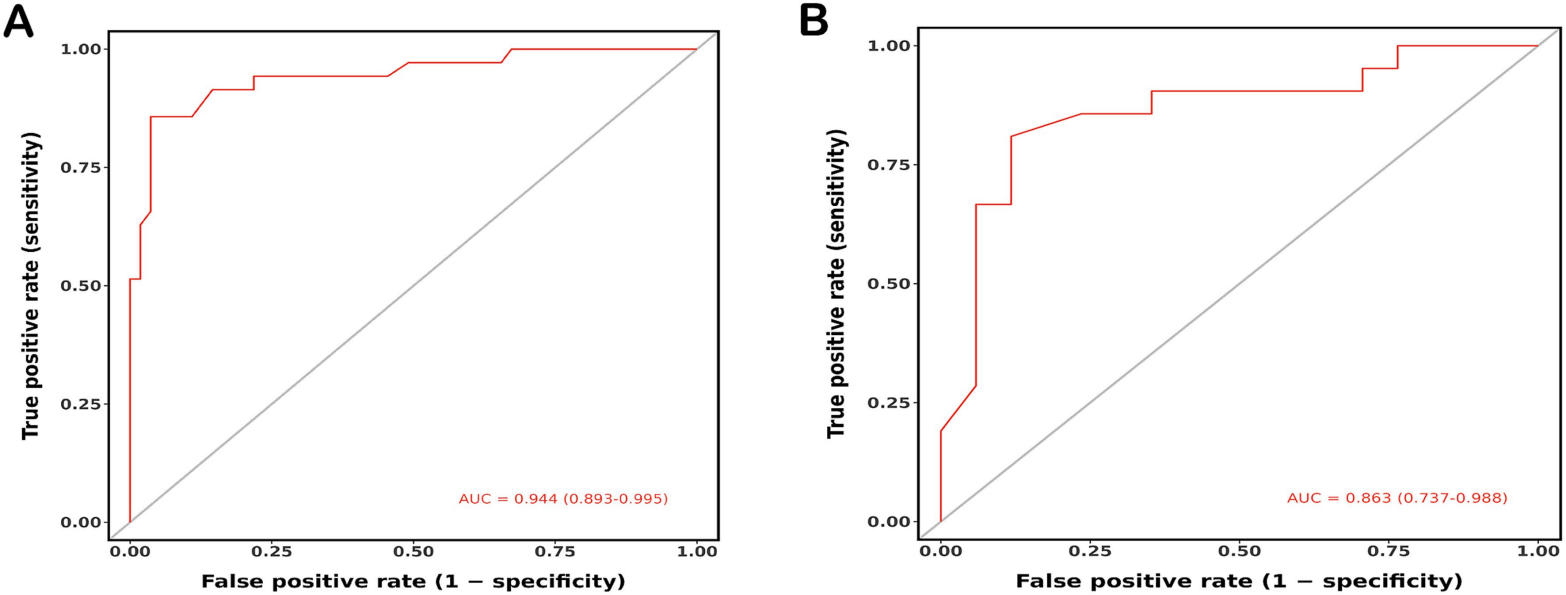
ROC analysis of nomogram for predicting the poor prognosis of MMD patients after bypass treatment in raining **(A)**, validation **(B)** cohorts.

**Figure 5:**
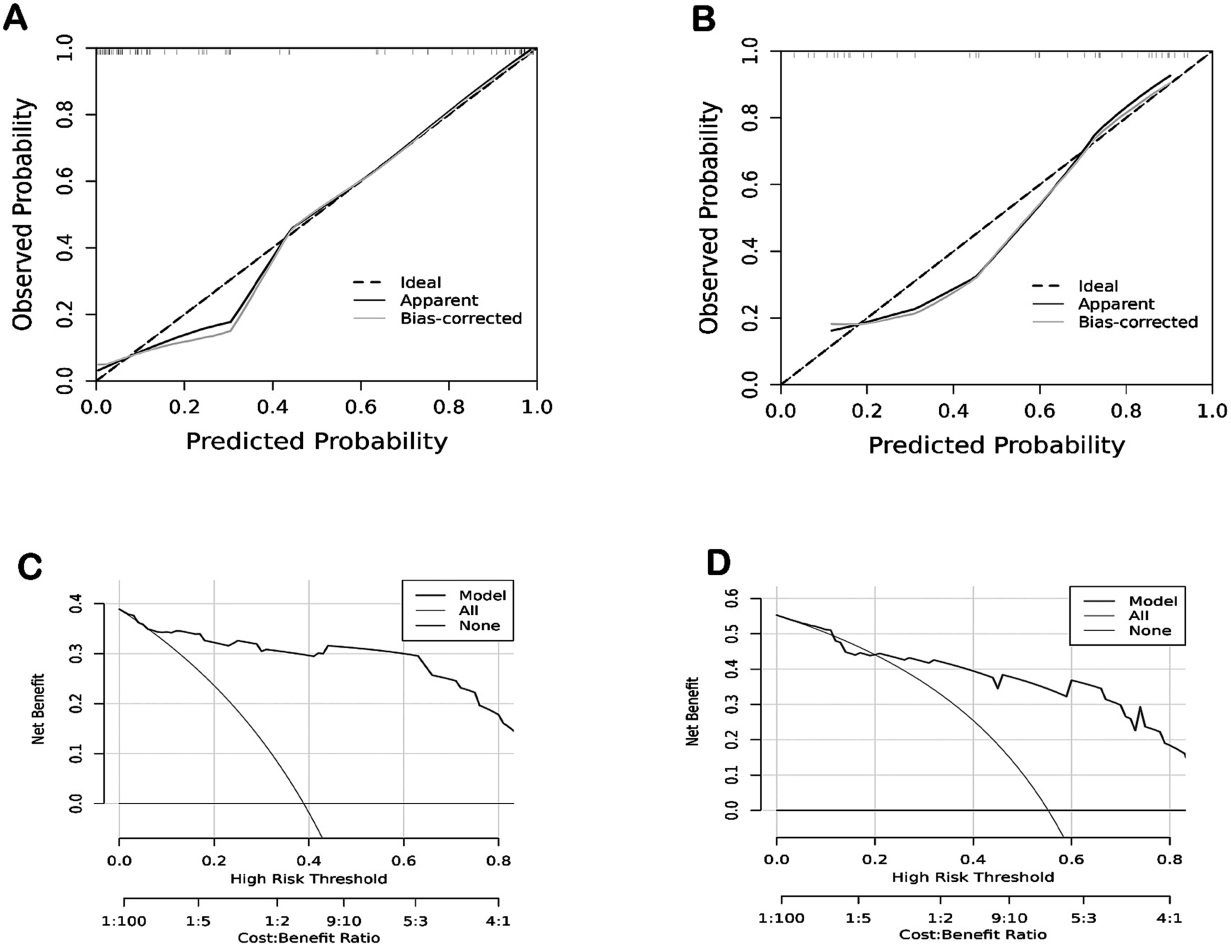
Calibration curves of t analysis of the nomogram model in the training **(A)**, validation **(B)** cohorts. Decision curves of the nomogram model in the training **(C)**, validation **(D)** cohorts.

Based on the results of multivariate logistic regression analysis, preoperative ASL-ASPECTS, admission mRS, ivy sign, and Houkin’s grade >2 were used to develop the nomogram prediction model (Figure 3). The AUCs for nomogram model for predicting poor prognosis in the training and validation cohorts 0.944 (95% confidence interval [CI]: 0.893–0.995) and 0.863 (95%CI: 0.737–0.988), respectively (Figures 4A,B). The calibration curve analysis showed that the efficacy of nomogram model in the training group was fine (Figures 5A,B). The DCA showed that a net benefit could for threshold probabilities of 0.1–0.8 in the training cohort and 0.2–0.8 in the validation cohort (Figures 5C,D).

Ten-fold cross-validation showed that the AUC values of nomogram prediction model for poor prognosis for all MMD patients were 0.918.

**Figure 6:**
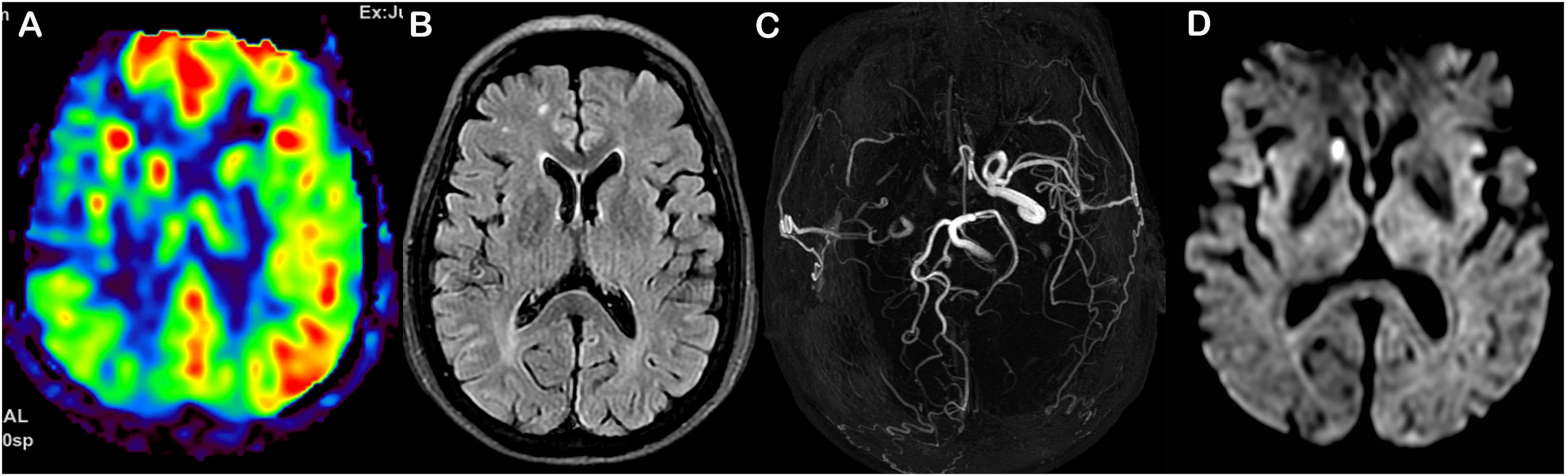
Representative MMD case with poor prognosis **(A–D)**. Female, 60 years old, with an admission mRS score of 2, and an mRS score of 3 after 1 year. T2-FLAIR showing ivy sign **(A)**; Preoperative ASL-ASPECT score was 4 **(B)**. Houkin score was 6 at 1 week postoperatively, and MRA staging was stage 3 **(C)**; DWI showing acute/subacute infarction in the right caudate nucleus **(D)**. The nomogram score was 130, with a probability of poor prognosis of 0.90.

**Figure 7:**
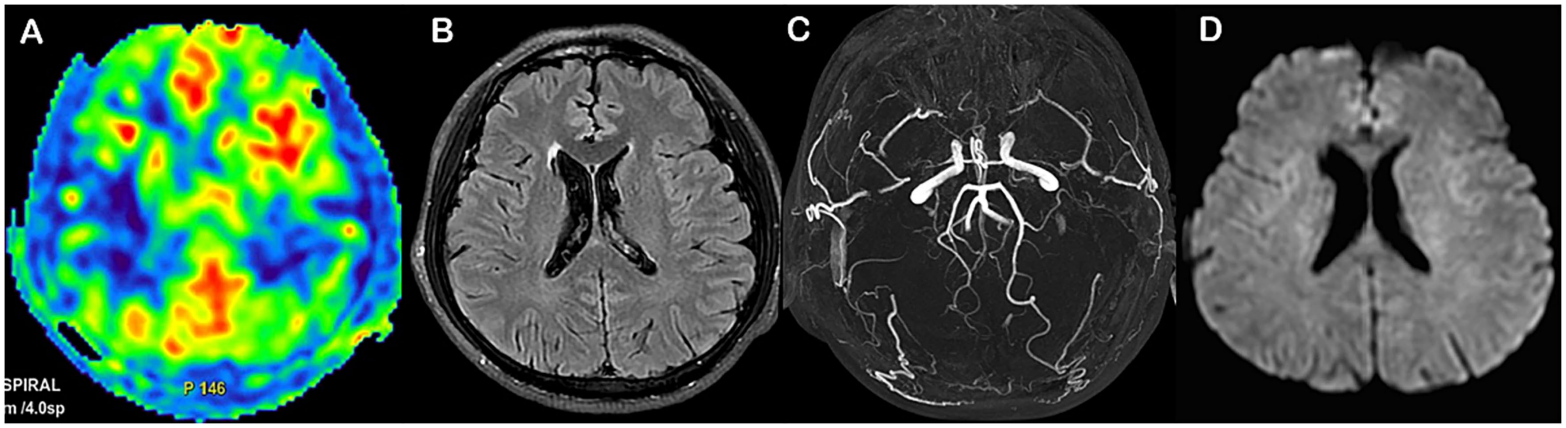
Representative MMD case with good prognosis **(A–D)**. Male, 50 years old, with an admission mRS score of 1, and the 1-year mRS score was 0. FLAIR image showed no ivy sign **(A)**; A Preoperative ASPECTS score was 7 **(B)**; Postoperative Houkin score was 3 at 1 week, and MRA staging was stage 2 **(C)**; Follow-up DWI showed no high signal **(D)**. The nomogram score was 25, with a probability of poor prognosis < 0.1.

Representative patients with MMD with poor and good prognoses are shown in Figures 6, 7, respectively.

## Discussion

This retrospective study identified significant factors associated with poor prognosis 1 year after surgical revascularization in patients with MMD. Our results demonstrated that preoperative ASL-ASPECTS, admission mRS score, ivy sign, and Houkin grade were significantly associated with postoperative outcomes. Based on these factors, we also developed and confirmed the performance of a practical nomogram. Our findings suggest that ASL perfusion metrics and vascular signs from conventional MRI should be incorporated into postoperative prognosis assessment in patients with MMD. The proposed model offers a quantitative tool to improve the accuracy and efficiency of poor prognosis risk stratification following bypass surgery in patients with MMD.

An important finding from this study is that ASL-ASPECTS may be a potential prognostic imaging biomarker following surgical revascularization in patients with MMD. While previous studies have linked ASL-ASPECTS with outcomes in patients with ischemic stroke (15, 17), to the best of our knowledge, no similar investigation has been reported for MMD in the English literature. The underlying mechanism by which ASL-ASPECTS contributes to the prognosis prediction in MMD likely relates to the pathological bases of MMD, including endothelial hyperplasia and abnormal smooth muscle cell migration, leading to vascular stenosis and collateral vascular network formation. The ASL-ASPECTS system indirectly reflects the effects of these vascular diseases by quantifying the changes in cerebral hemodynamics. A key novelty of the present study is the integration of ASL-derived CBF with regional cerebral damage based on the ASPECTS score. This combined approach helps overcome the limitations of relying on ASL-CBF metrics alone for prognostic assessment. Compared with DSC-MRI and CTP, ASL-ASPECTS was chosen for post-MMD treatment perfusion assessment for its several advantages, including the absence of the use of exogenous contrast agents, the absence of contrast agent side effects, and the absence of ionizing radiation. Thus ALS-ASPECTS was more suitable for the frequent evaluation of cerebral perfusion in patients with MMD before and after surgical revascularization treatment. However, due to the relatively small sample size in this study, future large-scale cohort studies are needed to validate the stability and predictive value of ASL-ASPECTS for long-term prognosis in MMD.

The results of the present study confirmed the prognostic value of MRA vascular grading following surgical revascularization in patients with MMD. Unlike previous studies, the strength of the present study is the use of postoperative MRA grading rather than preoperative assessment. Prior research has suggested that the predictive value of MRA grading in MMD may depend on the surgical approach (18). Specifically, the outcomes of patients undergoing direct revascularization were less associated with Suzuki stage, whereas the prognosis of those undergoing indirect revascularization was more strongly associated with Suzuki stage. In the present study, postoperative MRA staging effectively predicted prognosis and may mitigate the confounding effects of surgical technique.

In the present study, postoperative Houkin grade >2 was significantly associated with poor prognosis. This may indicate more advanced pathological state, with increased smooth muscle cell proliferation and intimal fibrosis in the vascular wall, which may inhibit postoperative angiogenesis (19). However, preoperative vascular grade and collateral circulation after revascularization show no significant in children with MMD, possibly due to increased vascular plasticity in children (18, 20). This study included only three pediatric patients with MMD, which limited the ability to thoroughly assess age-related differences in prognosis. As age was not identified as an independent prognostic factor, pediatric and adult patients were analyzed together. Future studies with larger pediatric cohorts are warranted to enable more robust evaluation of potential age-specific prognostic patterns.

Overall, the findings of the present study confirmed that the prognostic value of vascular grade after revascularization was comparable to preoperative assessments. Postoperative MRA offers a non-invasive method to dynamically monitor vascular changes and may be for MMD diagnosis and treatment, as well as postoperative management. Future studies should compare the prognostic significance of pre- and post-operative vascular grading in both adult and pediatric MMD populations.

The ivy sign on preoperative MRI was a significant factor for poor prognosis within 1 year following bypass surgery in patients with MMD in the present study, consistent with previous reports (21). However, contrary to previous studies, we did not distinguish the ivy sign in the ipsilateral from the contralateral cerebral hemisphere, suggesting that the presence of ivy sign on either the left or right hemisphere may be associated with poor prognosis. The pathological mechanism of ivy sign is the slow flow of leptomeningeal collaterals or leptomeningeal vasodilation caused by decreased CBF, suggesting an increased burden of leptomeningeal collaterals and decreased blood flow reserve in the ischemic area in patients with MMD (22).

Further studies by Li et al. showed the association of both ivy sign and Suzuki stage with postoperative collateral circulation (8). The present study also demonstrated that the combined use of Houkin grade and ivy sign predicted a poor prognosis, with a ROC curve AUC of 0.807, which was higher than the single index of either. These findings suggest a synergistic effect between the two indicators, highlighting their potential to improve prognostic accuracy when used in combination. Li et al. also explored the associations among ivy sign, Suzuki stage, and their correlation with postoperative collateral circulation (8). The present study also demonstrated that the combination of Houkin grade and ivy sign improved the prediction of poor postoperative prognosis in patients with MMD. These findings suggest a synergistic effect between the two indicators, and their combined use may improve prognostic accuracy in clinical practice.

Regarding clinical factors, the present study confirmed that admission mRS was associated with neurological prognosis within 1 year after bypass surgery. However, no significant correlation was observed between prognosis and age or hypertension. The association of admission mRS score >2 and poor prognosis in patients with MMD may be attributed to several factors (2, 23). First, posterior circulation involvement has been linked to higher mRS scores on admission and reduced functional improvement following bypass surgery, potentially due to compromised cerebrovascular reserve in the posterior circulation (24). Second, patients with MMD with higher mRS scores tend to have vascular damage and heightened inflammatory responses, including significantly elevated levels of interleukin-6 (IL-6) and tumor necrosis factor-alpha (TNF-α). These cytokines can promote endothelial apoptosis and accelerate arterial stenosis, exacerbating ischemic injury and potentially impairing postoperative angiogenesis, thereby contributing to poorer outcomes (25). Although previous studies have identified hypertension as an independent risk factor for unfavorable prognosis after revascularization in MMD (2, 26), the present study did not find hypertension to be a significant predictor of poor prognosis at 1 year, despite the higher rate of hypertension in the poor prognosis group. This discrepancy may be due to the small sample size and potential confounding factors in the present cohort. In previous studies, the functional prognosis for bilateral bypass surgery was typically assessed after the second operation. Consistent with this, the present study also used the timing of the second surgery as the baseline reference point. Distinguishing between unilateral and bilateral procedures in prognostic analyses may yield deeper insights into surgical outcomes and help clarify potential differences in recovery patterns.

Another strength of this study is its incorporation of not only preoperative neurological baseline scores (27) and vascular staging reflecting the burden of meningeal collateral circulation (28), but also vascular staging indicative of CBF compensatory capacity. As a result, the predictive performance of the combined model was superior to any other single-factor model. To our knowledge, this is the first study to objectively quantify both ASL-based perfusion metrics and conventional MRI features and to demonstrate their significant correlation with clinical outcomes following surgical revascularization in patients with MMD. This approach may improve our understanding of postoperative MMD prognosis and facilitate standardized, objective comparisons across future studies and patient populations.

This study has several limitations. First, as a retrospective study with small sample size from single-center, it is subject to inherent biases, including selection bias; only patients with available ASL perfusion imaging and conventional MRI were included, typically performed for specific clinical indications such as preoperative evaluation. However, the results in the validation cohort suggest the method’s stability and reliability, supporting its potential utility in future multi-center studies. The validation with larger cohorts, randomized trials, and external cohort validation will be needed in the future studies for improving generalizability. Secondly, the factors affecting the long-term prognosis of MMD are complex and diverse; to reduce variability, this study included only patients who underwent surgical revascularization and standardized baseline clinical and MRI characteristics. Third, the follow-up period was limited to 1 year, indicating the need for longer follow-up studies on disease progression and function outcomes. Despite this, meaningful insights were gained that may guide future prognosis research. Fourthly, the image analyses, including ROI, ASL-ASPECTS scoring, and Houkin’s grade, were performed manually, which may limit efficiency and introduce variability. Nonetheless, this study ensured consistency through pre-analysis training and consultation with a senior radiologist. Future incorporation of automated image segmentation and scoring methods could further improve the accuracy and efficiency of imaging assessments in patients with MMD. Lastly, ASPECTS score is the method for evaluating MCA ischemia. The ischemic stroke in MCA region in MMD patients can lead to contralateral hemiplegia, sensory disturbance, hemianopia and aphasia, which would affect the quality of life of patients obviously. However, the ASPECTS score does not take into account the fact that MMD is often involve ACA. Thus, relying solely on the ASL-ASPECTS score may not provide a comprehensive assessment of cerebral perfusion deficits in these patients. Therefore, the influence of abnormal perfusion in the ACA region on the functional prognosis after MMD bypass surgery should be evaluated in the future.

## Conclusion

In this study, lower preoperative ASL-ASPECTS, the presence of ivy sign, and a postoperative Houkin grade >2 based on ASL perfusion and conventional MRI were identified as independent predictors of poor prognosis in patients with MMD following surgical revascularization. Moreover, the nomogram incorporating these factors in combination with higher admission mRS scores demonstrated strong predictive performance in both the training and validation cohorts, suggesting the model’s stability and potential clinical utility. The model demonstrates excellent performance in our internal validation, though further external validation is warranted. Further multicenter studies with larger cohorts are warranted to further validate these findings and enhance generalizability.

## Data Availability

The datasets presented in this article are not readily available because the data that support the findings of the study are available from the corresponding author upon reasonable request. Requests to access the datasets should be directed to quanguanmin@hebmu.edu.cn.

## Footnotes

1
https://www.mstata.com/

## Glossary

ACA: Anterior cerebral artery
ASL: Arterial spin labeling
ASL-ASPECTS: Arterial spin labeling Alberta stroke program early CT score
AUC: Area under the curve
CBF: Cerebral blood flow
CI: Confidence interval
CT: Computed tomography
DCA: Decision curve analysis
FLAIR: Fluid-attenuated inversion recovery
ICA: Internal carotid artery
IQR: Interquartile range
MCA: Middle cerebral artery
MMD: Moyamoya disease
MRI: Magnetic resonance imaging
MRA: Magnetic resonance angiography
mRS: Modified Rankin scale
OR: Odds ratio
PCA: Posterior cerebral artery
PLD: Post-labeling delay
ROC: Receiver operating characteristic
ROI: Region of interest
T2-FLAIR: T2-weighted fluid-attenuated inversion recovery
TE: Echo time
TI: Inversion time
TOF: Time of flight
TR: Repetition time
WMH: White matter hyperintensity
